# Using temporal genomics to understand contemporary climate change responses in wildlife

**DOI:** 10.1002/ece3.9340

**Published:** 2022-09-16

**Authors:** Evelyn L. Jensen, Deborah M. Leigh

**Affiliations:** ^1^ School of Natural and Environmental Sciences Newcastle University Newcastle Upon Tyne UK; ^2^ Swiss Federal Research Institute WSL Birmensdorf Switzerland

**Keywords:** aDNA, genetic erosion, historical DNA, hybridization, local adaptation, population genomics

## Abstract

Monitoring the evolutionary responses of species to ongoing global climate change is critical for informing conservation. Population genomic studies that use samples from multiple time points (“temporal genomics”) are uniquely able to make direct observations of change over time. Consequently, only temporal studies can show genetic erosion or spatiotemporal changes in population structure. Temporal genomic studies directly examining climate change effects are currently rare but will likely increase in the coming years due to their high conservation value. Here, we highlight four key genetic indicators that can be monitored using temporal genomics to understand how species are responding to climate change. All indicators crucially rely on having a suitable baseline that accurately represents the past condition of the population, and we discuss aspects of study design that must be considered to achieve this.

## Graphical abstract

Population genomic studies that use samples from multiple time points (“temporal genomics”) are uniquely able to make direct observations of change over time, and are critical to documenting how species are responding to climate change. Here, we highlight some key genetic indicators that can be monitored using temporal genomics to understand how species are responding to climate change: genetic erosion, spatiotemporal population structure, hybridization, and adaptation.

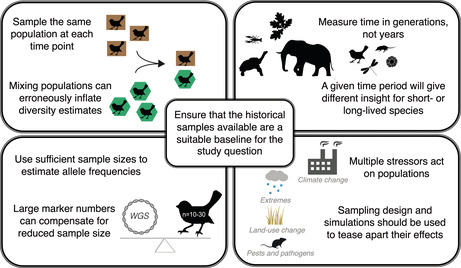

## 1 INTRODUCTION

The global climate is changing and population sizes of wild species are declining (WWF, 2020). Contemporary maladaptation, where the local genotypes of many species are no longer producing phenotypes optimally adapted to the environment they are in, is contributing to these population declines (Diamond & Martin, 2020). To counter this maladaptation and maintain fitness, many species have begun to respond to their changing climate through phenotypic plasticity, range shifts, and adaptation (Hughes, 2000). Characterizing how species are responding to climate change is now critical for biodiversity monitoring and conservation but disentangling the processes occurring using genomic data from a single snapshot in time is complex (Waldvogel et al., 2020). Population genomic studies that analyze time series of samples (hereafter, “temporal genomics”) can directly detect and quantify any change in genetic diversity, allele frequencies, or population structure over the sampling period, facilitating the detection of climate change responses.

Due to the valuable genetic information obtained, temporal genomics is increasing in popularity and demand (Habel et al., 2014), particularly because appropriate conservation measures are dependent on ongoing responses to climate change. For example, assisted gene flow may accelerate the dispersal or movement of pre‐adapted genes, but can also counteract the process of adaptation by disrupting ongoing local beneficial allele frequency shifts (Aitken & Whitlock, 2013). The effective genetic monitoring and safeguarding of genetic diversity mandated by the Convention on Biological Diversity's Post‐2020 Global Framework (Convention on Biological Diversity, 2021) are also dependent on temporal genomics (or genetics) as this pertains to the maintenance and restoration of genetic diversity, which can only be shown with temporal data.

**FIGURE 1:**
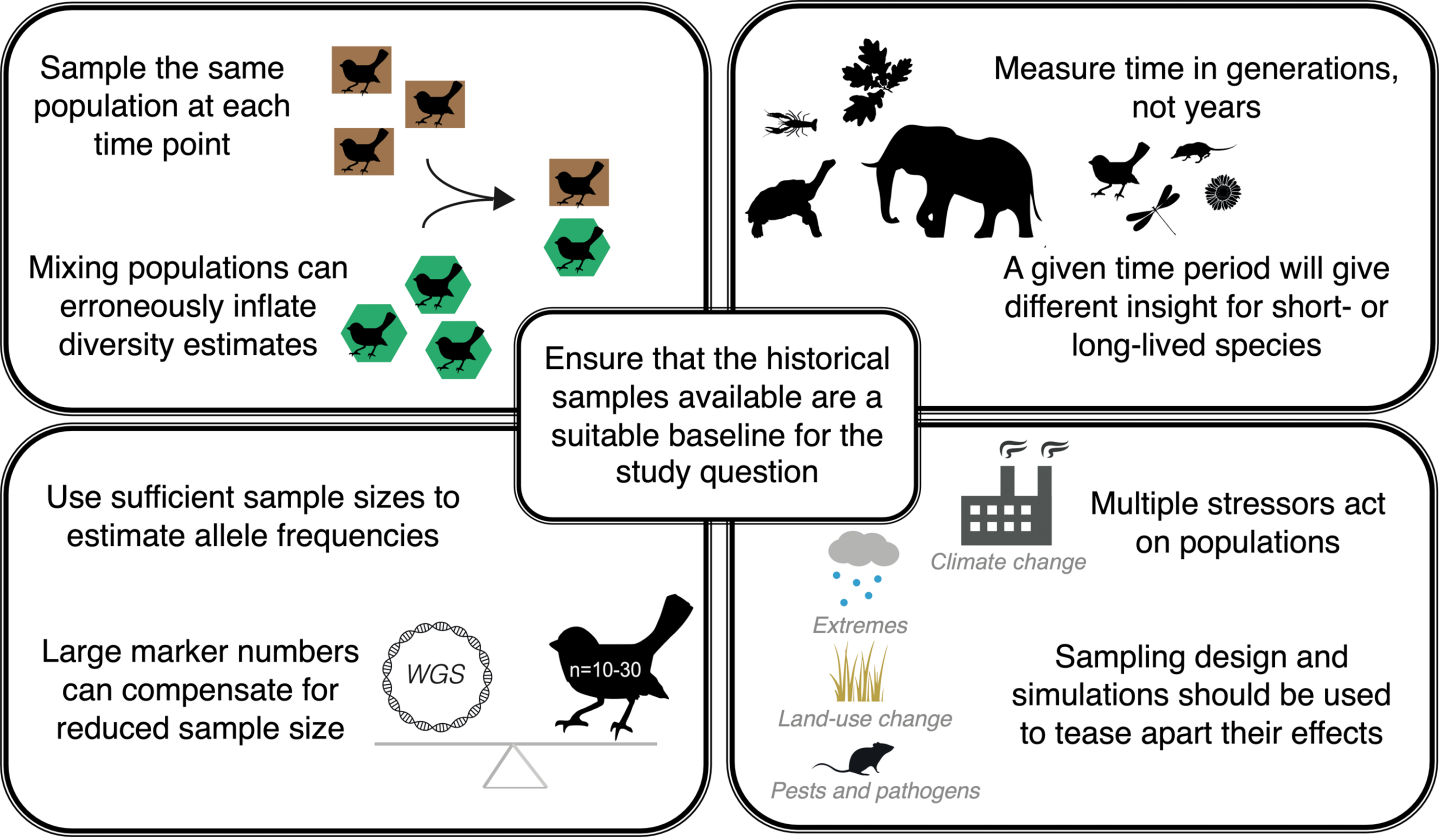
Some of the key considerations for temporal population genomic study design when examining climate change effects.

Here, we describe some of the challenges and considerations for a successful temporal genomic study (Figure 1), then present four key genetic climate change response indicators for which temporal analyses provide unique insights or increased analytical power. We stress the importance of a good baseline for each indicator given its critical role in all temporal inferences and highlight common misinterpretations that poor baselines can cause.

## 2 CHALLENGES AND CONSIDERATIONS FOR TEMPORAL POPULATION GENOMIC STUDIES

Obtaining a good baseline is the largest challenge for any temporal genomics study. An ideal temporal genomics study will have historical samples that accurately represent the baseline genetic variation in a population. This requires historical samples collected before the stressor of interest began and sufficient sample numbers to obtain accurate estimates of historical allele frequencies.

Poor quality of historical DNA is the most widely known limitation, but ancient DNA techniques are quickly advancing (e.g., optimized DNA extraction (Grealy et al., 2019), single‐stranded DNA library preparation (Troll et al., 2019), and hybridization capture (Schmid et al., 2017)) and enabling high‐quality genome‐wide data to be obtained even from the most challenging specimens (Straube et al., 2021) and time points deep in history (van der Valk et al., 2021). Other baseline limitations are less likely to be overcome with technical advances but are equally important, this includes the fact that historical specimens are simply not available for many taxa. The taxonomic and geographic biases in natural history collections mean that most species are not well represented (Meineke & Daru, 2021). If specimens exist, critical meta‐data such as date of collection and geographic location are often missing. Using such samples creates a high risk of conflating temporal and spatial patterns in genetic variation because the historical and contemporary samples are unlikely to originate from the same population. Finally, there is little guarantee that the historical specimens available are a representative sample of the population at the time (e.g., due to sex or age bias; Cooper et al., 2019). Thus, the reality is that most temporal studies will struggle to obtain an accurate baseline, and the effects of this need to be more widely considered (Box 1).

BOX 1 **Expected trends in genetic diversity**

When species are impacted by climate change, intraspecific genetic diversity will likely also be impacted. How genetic diversity will change is complex because values are dependent on random genetic drift, gene flow rates, and the population size maintained. In the figure below, the top panel shows a simplified trajectory of predicted changes in genetic diversity across a range of scenarios. Notably, trends in genetic diversity are unlikely to be linear because of genetic drift (Fuerst & Maruyama, 1986; Lacy, 1987). In declining populations experiencing genetic drift, because there are few copies of rare alleles they are lost easily and quickly, leading to elevated initial genetic diversity loss (measured by both heterozygosity and allelic diversity). After rare alleles are lost, the remaining alleles are all common and thus intrinsically harder to lose through genetic drift, slowing the rate of change and creating a non‐linear trend (solid line). However, some species, particularly those that retain a large effective population size, may deviate from this “L shaped loss” and show a more linear trend in genetic diversity change (dashed line). Species experiencing extreme climatic events, in contrast, could experience immediate genetic diversity change (dotted line) but this may not be permanent and could recover (not depicted). Expanding species with increased migration rates or species experiencing increased introgression rates may even experience an increase in genetic diversity (dot‐dash line). Points A, B, C, and D along the trajectories match the bottom panels that depict the population at different time points. In temporal genomic studies, the historical baselines used will have substantial impacts on the trends observed. For example, if samples from point B are used as a historical baseline and the true historical diversity is underestimated, the amount of genetic erosion will also be underestimated. Small sample sizes from the historical time point may also lead to an underestimation of genetic erosion, for example, if the hexagonal genotype in panel A is not sampled because it was historically rare.

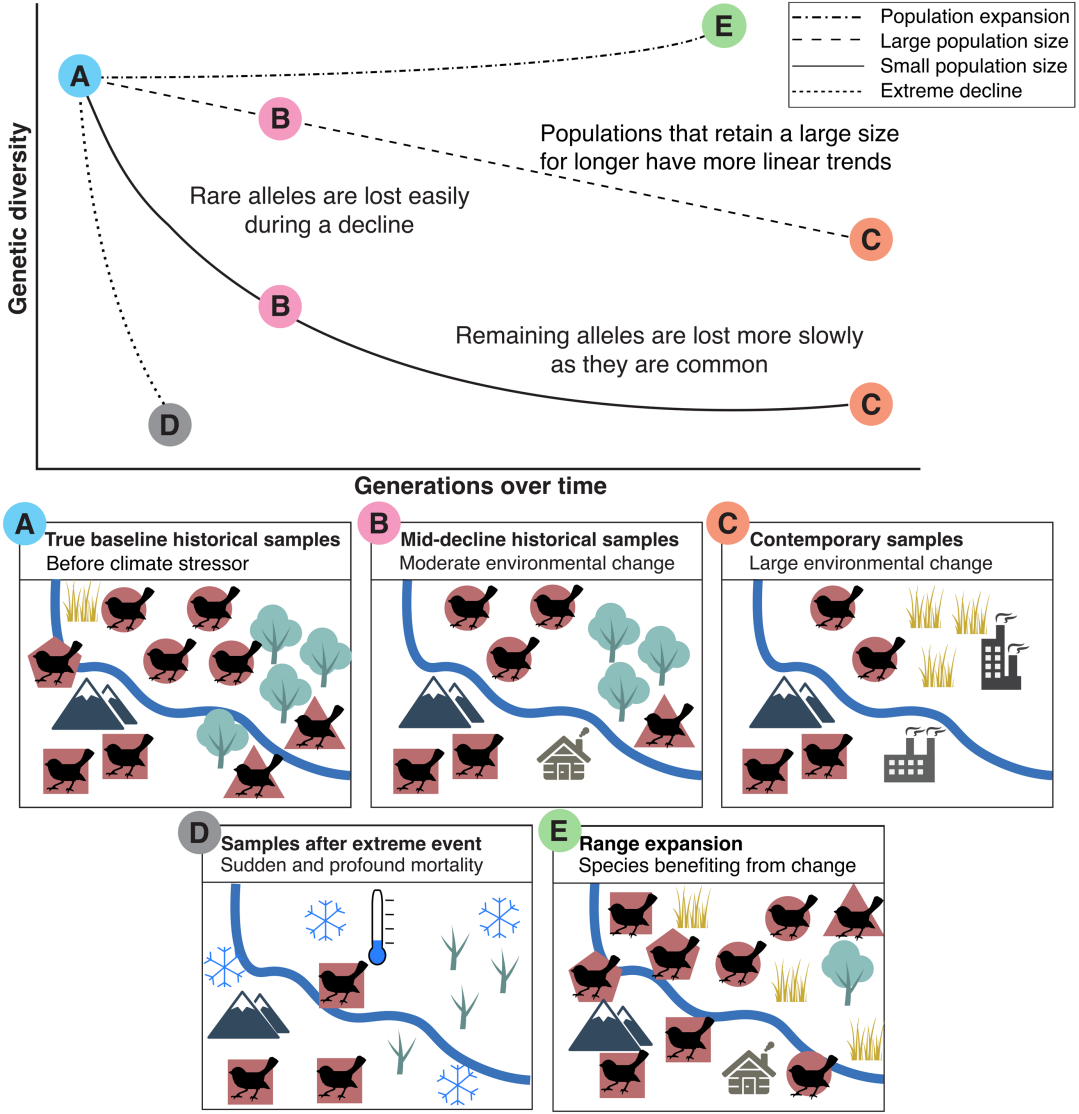

Temporal genomic studies with more recent baselines have several advantages and can still be highly informative. Specimens collected in the recent past (i.e., >1980s) may be readily available in good numbers in research collections and likely have meta‐data. Because many of the direct impacts of climate change began in the last century (Hawkins & Sutton, 2012), such recent baselines are going to be useful for climate change‐related studies despite the limited number of lapsed generations. This is particularly true for studies characterizing the effects of climate change‐mediated extreme weather events. In this scenario, a suitable baseline could be merely weeks old, only needing to occur prior to the event (see Box 2).

BOX 2 **Impacts of extreme events**

Extreme weather events (e.g., 2021 Texas blizzard; 2020 fires in Australia and California) are becoming increasingly common due to global climate change and have strong biodiversity impacts (Coleman & Wernberg, 2020). Evolutionary responses to extreme events have been shown to be contextual, dependent on the pre‐existing population and initial environmental conditions (Grant et al., 2017). Given a large number of existing population genetic datasets, scientists should consider revisiting populations after extreme events to observe their impacts and monitor population recovery. Campbell‐Staton et al. (2017) evaluated the impacts of an extreme cold event on green anole lizards (*Anolis carolinensis*) by revisiting a population that they had fortuitously sampled a few months prior to the event. Their study revealed rapid and strong selection on gene expression and cold tolerance. Similarly, in invasive species such as Burmese pythons (*Python bivittatus*), a rare freeze event leading to mass mortality was also used to identify directional selection on loci putatively associated with freeze tolerance (Card et al., 2018). Due to the tremendous strength of selection during these extreme events and their increasing frequency, these types of studies are now essential to fully understand climate change effects but will also yield many insights into the evolutionary process.

Besides causing possible strong selection, extreme events can also result in diversity loss due to high associated mortality, even if the event only occurs within a single generation. Temporal samples before and after a forest fire showed increased inbreeding and loss of genetic variation in two sympatric species of frog (Potvin et al., 2017). Post‐fire temporal monitoring demonstrated that one of the two species managed to fully recover. This example highlights that populations can recover from extreme events and revert to the pre‐extreme state. Care must be taken with contemporary sample collection in these new types of studies. Contemporary samples must be obtained immediately after an extreme event to fully quantify its impacts. Focal populations should also be repeatedly genetically monitored to establish recovery potential, identify if reversion has occurred, and ultimately identify the trans‐generational responses (Grant et al., 2017). Examining extreme event effects is an exciting avenue to study climate change responses in species with poor historical baselines and we would advocate for an increase in these types of studies.

Importantly, climate change effects are not occurring in isolation. Species are subject to a litany of other stressors (e.g., new diseases, habitat modification, and loss; Hof et al., 2011), as well as natural population genetic processes (e.g., mutation, genetic drift, migration, and hybridization). Natural climatic variation and cyclical processes (e.g., El Niño) are also occurring. These can impact allele frequencies in parallel with climate change effects, for example, cyclic seasonal changes in allele frequencies have been identified in wild populations of *Drosophila melanogaster* (Bergland et al., 2014). A carefully designed study with samples at multiple historic time points may be able to separate the specific effects of each stressor and process apart (notably, the inclusion of more recent historical baselines will be particularly informative on cyclical changes and species biology). However, directly attributing changes in populations to anthropogenic climate change may not always be possible. Such studies will still be informative, for example, for general biodiversity monitoring, and will offer insights into the evolutionary process. However, failure to pinpoint the exact driver of temporal change may result in inefficient conservation actions.

Ultimately, the results of temporal genomic studies must be interpreted with baseline limitations in mind to avoid erroneous conclusions. This is compounded by the fact that genetic processes are not necessarily linear (Box 1). Temporal genomic studies should also do basic assessments of the impacts of imperfect baselines (i.e., through simulations) before they destructively sample valuable historical specimens to determine which insights are possible or detectable given the samples available (Hoban et al., 2014). Databases such as GBIF (GBIF: The Global Biodiversity Information Facility 2022) are important tools that should be employed to scope out the extent of specimens available across institutions. Without this step, there is a risk of obtaining limited insights due to low power, and more seriously, incorrect conclusions with harmful conservation management decisions may be drawn.

## 3 KEY CLIMATE CHANGE RESPONSE GENETIC INDICATORS

It is important to remember that in a population at mutation–selection–drift equilibrium, no net change in genetic diversity is expected over time (Crow & Kimura, 1970); however, many drivers (e.g., population declines/altered migration rates/invasive species) can disrupt this equilibrium and lead to change. Temporal population genomic studies provide a powerful approach for monitoring by allowing the retrospective and empirical observation of genetic changes that have occurred.

### 3.1 Genetic erosion

Intraspecific genetic diversity is a core component of adaptive potential and therefore is instrumental in adaptive responses to climate change. While only a small fraction of the genome may be involved in an adaptation, this is hard to identify, and monitoring genome‐wide genetic variation is considered the best approach to conserving adaptation potential (DeWoody et al., 2021; Kardos et al., 2021). Observation of low genetic diversity (measured as heterozygosity or allelic diversity) at a single time point can have multiple drivers, each leading to very different conservation management strategies. Low genetic diversity can arise due to a population decline strengthening genetic drift, signaling a need for conservation management. Alternatively, the population may be naturally small and could have evolved with low levels of diversity (Liu et al., 2021). The key question is “has genetic diversity *changed*?” Characterizing this change, often termed “genetic erosion” when a loss has occurred, is one of the most frequent objectives of temporal genomic studies. However, studies directly examining whether genetic erosion has been caused by climate change itself (rather than habitat loss or invasive species) are currently rare. A now well‐known example is from populations of *Tamias alpinus*, an alpine specialist chipmunk, in Yosemite National Park. It has undergone upslope range retractions due to increases in minimum winter temperatures, and analysis of temporal samples from the 1910s and present found genetic erosion had occurred because of this (Rubidge et al., 2012).

Genetic erosion is commonly incorrectly considered to occur only in species of conservation concern or over‐harvested commercial species. However, trends of genetic diversity loss are visible even in species of Least Concern (Leigh et al., 2019). The role of climate change in this loss is currently unclear and future studies are needed to explore this where possible. While it will be challenging to disentangle driver effects, gaining a better understanding of climate change's role in this loss is important for predicting species extinction risks, population trends, and future distributions. Predictions have been made about which regions of the globe are likely to experience genetic diversity loss due to climate change (Theodoridis et al., 2021), and temporal monitoring of genetic diversity will be essential to assess the accuracy of such predictions.

Accurately observing and correctly interpreting any changes in levels of genetic diversity is particularly sensitive to the baseline used. Ensuring that the temporal samples come from the same genetic population is critical, as natural spatial variation in diversity can easily be incorrectly attributed to temporal variation (Paz‐Vinas et al., 2021). Due to the potentially non‐linear decline in diversity in newly small populations, generic erosion is also likely to be underestimated if the baseline samples were collected after the onset of population decline (discussed in Box 1). Furthermore, erosion of allelic richness and heterozygosity are unlikely to occur at the same rate (e.g., Crow & Kimura, 1970; Schmid et al., 2018), as these measures have different sensitivities to the degree and length of population decline and to rare alleles (Cornuet & Luikart, 1996). Baseline sample sizes must also be sufficient to capture historical allele frequencies accurately and observe a rare variation. A poor baseline can easily lead to incorrect genetic trends, for example, a limited historical baseline spread across a large spatial‐temporal distribution for the pale‐headed brush finch (*Atlapetes pallidiceps*) is thought to have masked signals of severe genetic erosion (Hartmann et al., 2014).

### 3.2 Spatiotemporal population structure

Climate change is altering species ranges (see Box 3), which in turn is changing population connectivity and thus structure (Chen et al., 2011). Temporal sampling is the only way to reveal changes in population structure over time. If temporal samples truly originating from the same location are differentiated (i.e., *F*_ST_ values are significantly above 0), several scenarios may have played out: (a) local extinction and recolonization by a different lineage occurred (i.e., lineage replacement); (b) increased gene flow with other populations caused large shifts in allele frequencies; or (c) the population became isolated and experienced strong genetic drift. Climate change could drive any of these three scenarios, and understanding what has occurred can provide valuable insights into how species are responding and how to manage them.

BOX 3 **Dynamic species ranges**

As climate shifts, the geographic locations where species are found are changing. Some species' ranges are expanding, while others are contracting and others shifting to follow their historical climatic niche (Chen et al., 2011). Of particular concern are alpine species that can only retreat so far up‐slope (Freeman et al., 2018). However, as winters warm, tropical species whose ranges are currently limited by their lack of cold tolerance are also undergoing range shifts by expanding into previously temperate regions (Osland et al., 2021) and boreal species are extending into the arctic (Descamps & Strøm, 2021). Other species that are gaining notoriety as “winners” of climate change due to ongoing expansions are pest insects, such as the mountain pine beetle (Sambaraju & Goodsman, 2021) and disease vectors, including *Aedes aegypti* mosquitoes (Iwamura et al., 2020). An array of genetic processes can accompany range shifts (genetic erosion, increased or new hybridization events, allele surfing, lineage replacement, and adaptation) and temporal genomics is an important tool for characterizing change, thus improving predictions for future further shifts. Studies that integrate the investigation of temporal and spatial population genetic processes are likely to be particularly fruitful, as demonstrated by Popa‐Báez et al. (2020) in their study of the Queensland fruit fly (*Bactrocera tryoni*). They used temporal samples from the center and margins of the native range and a broad spatial sample of the recently invaded range to investigate the range expansion process.

The population genetic processes at range margins are complex, potentially involving serial founder effects, large changes in gene flow, and altered selective environments (Angert et al., 2020; Sexton et al., 2009). Range margins are often genetically depleted relative to the range core (Eckert et al., 2008). Whether this genetic depletion hinders adaptation during range shifts (due to deleterious alleles “surfing” to high frequency) or facilitates it (through rapid fixation of traits that are adaptive) is unclear (Hallatschek & Nelson, 2010). Temporal studies of wild species shifting their ranges under climate exchange will help address this question in the near future.

Opportunistic sequential temporal samples from Atlantic cod (*Gadus morhua*) have shown population‐specific patterns of lineage stability in the face of climatic shifts and over‐fishing. In some cases, little differentiation is observed, indicating populations have remained highly stable since the 1900s, while other populations have clearly gone extinct and been replaced by another lineage (Therkildsen et al., 2013). This example also shows the array of climate change responses that can occur in a single species, which is essential conservation information that will need to be reflected in management plans. In such studies, baseline considerations remain important, and ideally, historical samples from the different populations should be from a similar time period to avoid confounding the impacts of various stressors and natural changes in population structure over time.

### 3.3 Adaptation

Phenotypic plasticity and latitudinal/altitudinal range shifts have limitations, thus finding recent adaptations in climate change that threatened species is a key goal for conservation researchers. For climate change winners, the new habitats and stressors they encounter will also foster new adaptations. Identifying adaptation to local environmental conditions has thus become almost routine in single time point population genomic studies, in large part due to the huge potential for genome‐wide markers to identify regions putatively under selection (Hoban et al., 2016). While most studies use single time point data and samples across environmental gradients (Lotterhos & Whitlock, 2015), temporal samples have shown considerable promise for improving study power since they directly measure changes within the population and do not rely on space‐for‐time assumptions about population trajectories.

Temporal samples have been used to directly observe selection‐mediated allele frequency change over generations in wild populations (e.g., in response to Tasmanian Devil facial tumor disease; Epstein et al., 2016), and conducting such studies in climate change contexts is a promising avenue for research. For example, the chipmunk, *T. alpinus*, discussed above shows signs of directional selection at a candidate gene that may be associated with the physiological stress of climate change (Bi et al., 2019). Excitingly, the increased power of temporal selection detection methods may extend to bottlenecked species which can have an insurmountable number of false signals of selection due to their intrinsic history of strong genetic drift (Leigh et al., 2021). The selective sweeps that we expect due to rapid climatic adaptation or very strong climate‐mediated selection can also lead to genetic diversity loss due to hitchhiking or reduced effective population size (e.g., Atlantic silversides, *Menidia menidia*; Therkildsen et al., 2019). Consequently, temporal adaptation studies are also essential to gain insight into drivers of genetic erosion, and investigations into the two processes should be done in parallel. Increasing recognition of the power of temporal samples to test for adaptation has led to the recent development of multiple new methods to harness such datasets. These methods have been comprehensively reviewed by Malaspinas (2016) and Dehasque et al. (2020). With the rapid pace at which time series of whole‐genome datasets are being collected, before long we expect these methods will begin to deliver valuable insights into how species are adapting to climate change.

### 3.4 Impacts on hybridization

Climate change‐derived rage shifts are leading to colonization of new habitats, resulting in formerly isolated species living in sympatry, which can result in increased hybridization or shifting hybrid zones. While hybridization is a natural process, it is problematic when it has occurred due to anthropogenic impacts (Rhymer & Simberloff, 1996). Climatic conditions play a large role in limiting the spread of invasive species, and many are expanding with the warming climate. Invasive rainbow trout (*Oncorhynchus mykiss*), for example, were present in North America for several decades but only recently began to expand and hybridize with native cutthroat trout. In this case, temporal samples tracking levels of admixture over a 30‐year period and modeling of climatic variables showed that decreases in spring precipitation and increases in summer stream temperature were facilitating the spatiotemporal spread of hybridization between the two trout species (Muhlfeld et al., 2014). Understanding the conditions under which hybridization started to occur is helpful for assessing the risk of other populations to this threat.

Historical samples may help identify if hybridization has changed in frequency or direction. However, obtaining an accurate historical estimate of the frequency of hybridization may be difficult. Hybrids could be over‐represented in historical collections if they presented unusual phenotypes that appealed to collectors or may not be represented at all due to random chance, potentially leading to a false conclusion that hybridization did not occur in the past. Thus, studies examining changes in hybridization must be cautiously conducted using as large a baseline sample as possible.

## 4 CONCLUSIONS

Long‐term ecological and environmental datasets have proven invaluable for informing conservation management and policy (Hughes et al., 2017), and population geneticists are in the enviable position of being able to newly generate such data by making use of historical specimens. Thus, we predict that the interest in temporal population genomics studies will continue to increase, particularly as monitoring of genetic biodiversity becomes part of the new post‐2020 Convention on Biological Diversity. While it is important to maximize the information gained from historical samples due to their value, it is also important to ensure the samples available are suitable for answering the questions of interest. Misinterpretation of the degree to which historical samples represent the baseline could lead to erroneous conclusions that negatively impact species conservation. Furthermore, directly linking observed changes in populations to climate change will remain a challenge. Nevertheless, well‐designed temporal genomic studies have huge potential to reveal how wildlife species are being impacted by climate change, assisting conservation and biodiversity monitoring.

## AUTHOR CONTRIBUTIONS

**Evelyn L. Jensen:** Conceptualization (equal); investigation (equal); visualization (equal); writing – original draft (equal); writing – review and editing (equal). **Deborah M. Leigh:** Conceptualization (equal); investigation (equal); visualization (equal); writing – original draft (equal); writing – review and editing (equal).

## FUNDING INFORMATION

Lib4RI ‐ Library for the Research Institutes within the ETH Domain: Eawag, Empa, PSI & WSL, funded the open access publication fees for this article.

## CONFLICT OF INTEREST

The authors declare that there is no conflict of interest.

## Data Availability

No data were produced.
